# Reduce Manual Curation by Combining Gene Predictions from Multiple Annotation Engines, a Case Study of Start Codon Prediction

**DOI:** 10.1371/journal.pone.0063523

**Published:** 2013-05-10

**Authors:** Thomas H. A. Ederveen, Lex Overmars, Sacha A. F. T. van Hijum

**Affiliations:** 1 Centre for Molecular and Biomolecular Informatics, Radboud University Medical Centre, Nijmegen, The Netherlands; 2 Netherlands Bioinformatics Centre, Nijmegen, The Netherlands; 3 NIZO Food Research, Kluyver Centre for Genomics of Industrial Fermentation, Ede, The Netherlands; 4 Top Institute Food and Nutrition, Wageningen, The Netherlands; Albert Einstein College of Medicine, United States of America

## Abstract

Nowadays, prokaryotic genomes are sequenced faster than the capacity to manually curate gene annotations. Automated genome annotation engines provide users a straight-forward and complete solution for predicting ORF coordinates and function. For many labs, the use of AGEs is therefore essential to decrease the time necessary for annotating a given prokaryotic genome. However, it is not uncommon for AGEs to provide different and sometimes conflicting predictions. Combining multiple AGEs might allow for more accurate predictions. Here we analyzed the *ab initi*o open reading frame (ORF) calling performance of different AGEs based on curated genome annotations of eight strains from different bacterial species with GC% ranging from 35–52%. We present a case study which demonstrates a novel way of comparative genome annotation, using combinations of AGEs in a pre-defined order (or path) to predict ORF start codons. The order of AGE combinations is from high to low specificity, where the specificity is based on the eight genome annotations. For each AGE combination we are able to derive a so-called projected confidence value, which is the average specificity of ORF start codon prediction based on the eight genomes. The projected confidence enables estimating likeliness of a correct prediction for a particular ORF start codon by a particular AGE combination, pinpointing ORFs notoriously difficult to predict start codons. We correctly predict start codons for 90.5±4.8% of the genes in a genome (based on the eight genomes) with an accuracy of 81.1±7.6%. Our consensus-path methodology allows a marked improvement over majority voting (9.7±4.4%) and with an optimal path ORF start prediction sensitivity is gained while maintaining a high specificity.

## Introduction

The accurate annotation of bacterial genomes is essential to apply sequence data in many (bio)medical research topics such as microbiology, immunology and infectious diseases [1], [2]. It is required for a better understanding of the biology of bacteria as it involves identification of genes and subsequent proteins, regulatory networks and pathways. In practice, genome annotation often starts with the submission of a genome sequence to online annotation services, also named automated genome annotation engines/pipelines, which will be referred to as AGEs throughout this manuscript [3]. The output of these services usually consists of *ab initio* predicted open reading frames (ORFs) with start-, stop positions and function predictions. Start- and stop codon prediction is usually performed by ORF calling software, such as GLIMMER [4], [5], GeneMark [6], [7] or Prodigal [8], implemented in these AGEs. Correctly predicting ORFs is essential; prediction of gene function, ribosomal binding sites, promoter mapping, and subcellular location are all dependent on correct start codon prediction. Subsequent functional annotation of ORFs involves many steps including BLAST-like [9] searches in existing databases such as RefSeq [10], Genbank [11] and SwissProt [12], or hidden Markov model screenings with Pfam [13] or FIGfams [14]. As AGEs consist of different prediction steps and associated parameters (Table S1), they can for a given genome suggest different ORF predictions [15]. AGEs not uncommonly provide incorrect annotation calls [16], [17] (according to our study, roughly 14%–58% of start codon predictions are incorrect; see below). This begs the question: which AGE to choose for my genome of interest? Next to choosing a particular AGE to annotate a genome of interest, majority voting has been suggested as a method to combine predictions from different ORF prediction algorithms [18]–[23]. However, one is unable to know which predictions are likely in need for manual curation and which are likely to be correct. Therefore, most predicted ORFs are manually curated for start- and stop codons and gene function [24]. In order to prioritize genes to be manually curated it would therefore be highly advantageous to allocate a level of confidence to every ORF prediction.

Here we studied the start codon prediction performance of different AGEs based on a total of twelve strains from different bacterial species with widely differing GC%. Eight of these genomes have GC% ranging from 35–52% and the remaining four genomes have more extreme GC%. The genome annotations of the twelve well-studied strains have been extensively (manually) curated and are therefore considered to be of high quality. We chose not to incorporate stand-alone ORF calling software (e.g. GLIMMER [4], [5], GeneMark [6], [7] and Prodigal [8]) as this work is meant as a practical case study and therefore focuses on AGEs, as these are commonly used in annotation efforts. As stop codon predictions are only rarely being predicted wrong by AGEs (see below) we present a novel method to combine the result of multiple AGEs in order to more reliably predict start codon locations. Our work-flow uses consensus predictions by specific combinations of AGEs in a particular order (or path). This path was quite conserved for the eight moderate GC% organisms (35–52%) under study but less with the more extreme GC% genomes. The order of AGE combinations is from high to low specificity, where the specificity is based on the start codon prediction performance of these AGE combinations on the individual genome annotations. Based on the eight moderate GC% genomes, this path allows us to correctly predict start codons for 90.5±4.8% of the genes in a genome with an accuracy of 81.1±7.6%. For each AGE combination we are able to derive a novel so-called projected confidence value, which is the average specificity of ORF start codon prediction based on the eight genomes. This projected confidence value allows pinpointing ORFs of which the start codon is likely notoriously difficult to predict. We hypothesize that the proposed concept can also be applied to the prediction of stop codons and importantly gene function, allowing a researcher to focus resources by manually curating fewer genes.

## Results

**Figure 1:**
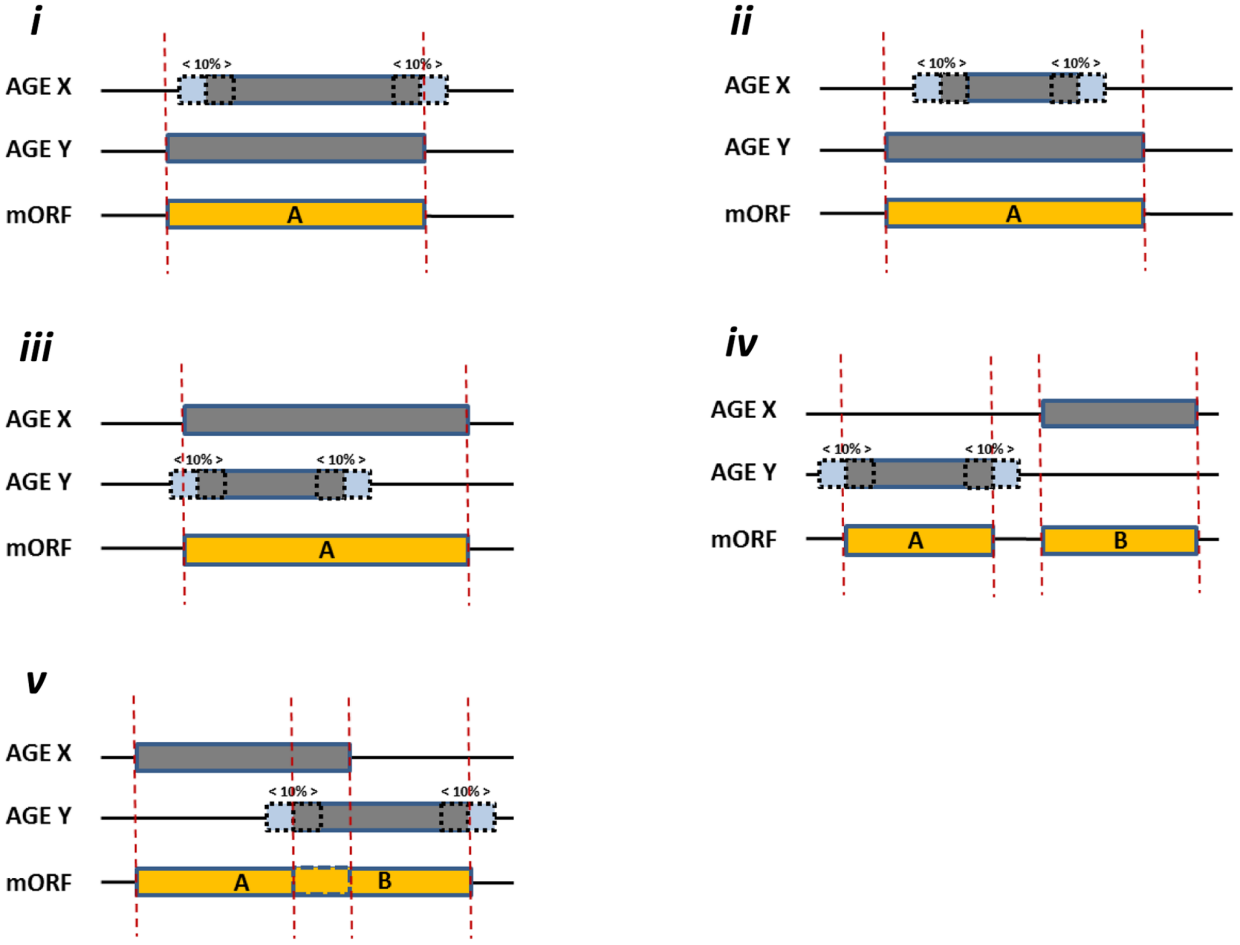
Assignment of meta ORFs. ORFs that contain start- (or stop) codon coordinates which are located in 10% proximity (of the length of the shortest ORF in the set of two ORFs being compared) to its neighboring ORF’s start- (or stop) codon coordinate, were grouped into the same mORF prediction. Gray boxes: predicted ORFs, with 10% margins of their length up- and downstream indicated by blue, dashed boxes. Yellow boxes: reference genome ORFs. Here, five exemplary mORF allocations are illustrated: (**i**) The stop codon coordinate of the suggested ORF provided by AGE_Y_ matches the ORF stop coordinate of that suggested by AGE_X_. Therefore, these two ORFs are grouped into the same mORF (mORF A). (**ii**) The ORF predicted by AGE_X_ falls within the ORF predicted by AGE_Y_. Therefore, these two ORFs are grouped into the same mORF (mORF A). (**iii**) The start codon predicted by AGE_x_ falls within the 10% boundary of the start codon predicted by AGE_y_. Therefore, these two ORFs are grouped into the same mORF (mORF A). (**iv**) The predicted ORF by AGE_x_ contains start- and stop codons both located outside the 10% boundary to the start- and stop codons of the ORF predicted by AGE_y_. Therefore, these two ORFs are assigned different mORFs (mORFs A and B). (**v**) Comparable to iv, the predicted ORF by AGE_x_ contains start- and stop codons located outside the 10% boundary to the start- and stop codons of the ORF predicted by AGE_y_. Even though there is small overlap between these two ORFs (dashed line), they are assigned different mORFs (mORFs A and B) because they exceed the 10% limit. Note that predicted ORFs by the fictive AGEs X and -Y must have the same orientation to be assigned to the same mORF.

**Figure 2:**
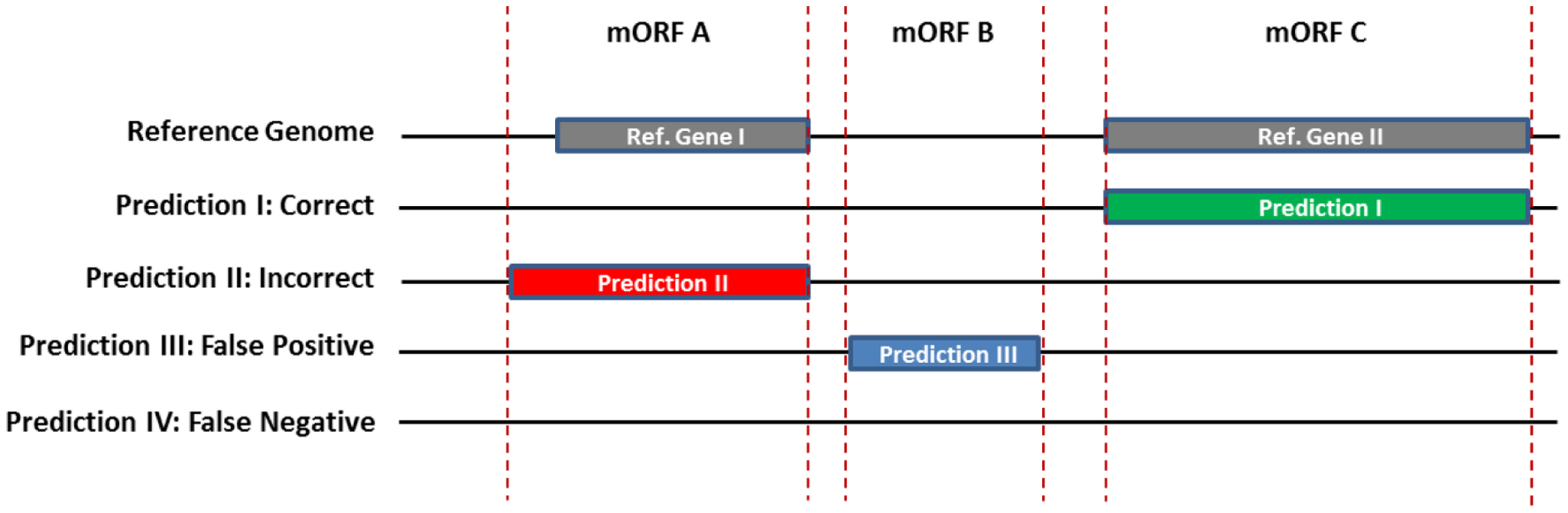
Classification of AGE gene predictions. Gray: reference genes from a bacterial reference genome with which the AGE predictions are compared. I (green): correct AGE prediction with matching coordinates. II (red): “incorrect” predictions with an incorrect start- and/or stop codon, but belonging to the same mORF. Note that the majority of stop codons were correctly assigned (Fig. S1B). III (blue): false positive ORFs predicted by an AGE and not present in the reference genome. IV: false negative predictions were allocated to AGEs that failed to predict a reference ORF.

**Figure 3:**
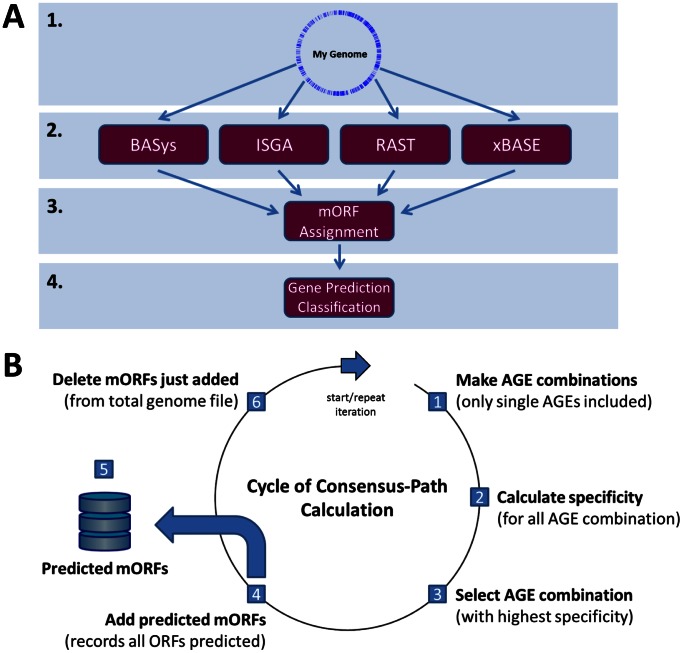
The methodology of comparative annotation and a step-by-step description for the consensus-path. **A.** In our methodology, a single genome of interest (**1**) is first uploaded to the four different AGEs (**2**) for ORF prediction and annotation (see also Table 1). After receiving all predictions from the respective AGEs, a mORF assignment is performed (**3**), as described in Figure 1. Finally, on this set of mORFs for the genome of interest, the AGE gene predictions are classified (e.g., correct and incorrectly predicted mORFs) (**4**) as described in Fig. 2. **B.** With the mORFs generated as described in A, a consensus-path calculation can be performed to find the sequential (combinations of) AGEs predicting the subset of mORFs under study with the highest specificity. This cycle (or iteration) consists of the following steps: (**1**) generating all possible AGE combinations (single AGEs only are also included) within the set of engines used (**2**) calculating for each possible AGE combination (or single AGE) its specificity for that subset of mORFs considered (see *formula 2* in the materials and methods). AGE combinations selected in the previous round are omitted in subsequent iterations. (**3**) The AGE combination (or single AGE) generating the highest specificity (hence, lowest error-rate) is selected. (**4**) These predicted mORFs are added to the (existing) list of predicted mORFs (**5**), for the genome of interest. (**6**) mORFs selected in (**4**) are removed from the mORFs file originally started with. The remaining mORFs are subjected to a next step of selecting the AGE (combination) with the highest specificity (**1**). This iteration is repeated until for a given genome no new mORFs are added to the prediction results in (**4**).

We studied the ORF start codon predictions by four AGEs (BASys, ISGA, RAST and xBASE; Table 1) for twelve genomes from well-studied strains of different bacterial species. This set of twelve genomes consists of eight genomes with a moderate GC% (percentage guanine-cytosine; moderate is defined as a range from 35–52%): *Escherichia coli* K12 MG1655, *Bacillus subtilis* 168, *Lactobacillus plantarum* WCFS1, *Lactococcus lactis* KF147, *Streptococcus pneumoniae* TIGR4, *Salmonella enterica* subsp. *enterica* serovar Typhi str. Ty2, *Neisseria meningitidis* MC58 and *Haemophilus influenzae* Rd KW20 (Table 2); and four genomes with a more extreme GC content: *Mycobacterium tuberculosis* H37rv, *Mycoplasma mobile* 163K, *Pseudomonas putida* KT2440 *and Streptomyces coelicolor* A3(2) (Table 2). In our analysis we evaluated all ORFs that were either predicted by an AGE or that were present in the reference genomes (Figs. 1 and 2 and see materials and methods). Below we explore, next to our consensus-path prediction method (Fig. 3), different alternative approaches to obtain accurate start codon predictions. The results for these alternative approaches are based on a representative set of four moderate GC% genomes: *E. coli* K12 MG1655, *B. subtilis* 168, *L. plantarum* WCFS1, *L. lactis* KF147 (Table 2). Next, we present the results of our consensus-path approach based on the above-mentioned twelve genomes.

**Table 1:** Automated genome annotation engines (AGE) used in this study.

Engine name (AGE)	Website of AGE	Reference
BASys	http://basys.ca/	Hemmerich et al., 2010
ISGA	http://isga.cgb.indiana.edu/	Aziz et al., 2008
RAST	http://rast.nmpdr.org/	Chaudhuri et al., 2008
xBASE	http://www.xbase.ac.uk/annotation/	Van Domselaar et al., 2005

The listed AGEs are commonly used pipelines for analysis and annotation of gene function and start- and stop codons.

**Table 2:** Genomes used in this study.

Strain (genome)	GC%	Genome size(Mb)	Gram (+/−)	Sequence date*/last update	NCBI accessionnumber	Number of annotated genes**
*Bacillus subtilis* 168	44	4.22	+	18-NOV-1997 20-JAN-2012	NC_000964.3	4262
*Escherichia coli* K12 MG1655	50	4.64	−	16-JAN-1997 11-JAN-2012	NC_000913.2	4235
*Haemophilus influenzae* Rd KW20	38	1.83	−	28-JUL-1995 15-OCT-2012	NC_000907.1	1715
*L. lactis* KF147	35	2.60	+	01-DEC-2009 21-NOV-2011	NC_013656.1	2605
*Lactobacillus plantarum* WCFS1	44	3.31	+	25-JUN-2001 21-NOV-2011	NC_004567.1	3128
*Mycoplasma mobile* 163K	25	0.77	−	13-APR-2004 01-APR-2010	NC_006908.1	661
*Mycobacterium tuberculosis* H37rv	66	4.41	−	13-SEP-2001 20-AUG-2012	NC_000962.2	4048
*Neisseria meningitidis* MC58	52	2.27	−	17-MAR-2000 19-JAN-2012	NC_003112.2	2122
*Pseudomonas putida* KT2440	61	6.18	−	08-APR-2002 27-SEP-2012	NC_002947.3	5424
*Streptomyces coelicolor* A3(2)	72	8.67	+	09-MAY-2002 19-JAN-2012	NC_003888.3	7833
*Salmonella enterica* subsp. *enterica* serovar Typhi str. Ty2	52	4.79	−	25-SEP-2002 24-OCT-2012	NC_004631.1	4448
*Streptococcus pneumoniae* TIGR4	40	2.16	+	29-JUN-2001 20-JAN-2012	NC_003028.3	2163

Listed is genome (sequence) data from the twelve reference strains used in this study (genome annotations as of the December 07^th^ 2012).

* According to GenBank (http://www.ncbi.nlm.nih.gov/genbank/) [11].

** Totaling both protein-coding and tRNA genes; no pseudogenes were included.

### ORF Predictions of the Four AGEs only Partly Overlap

**Figure 4:**
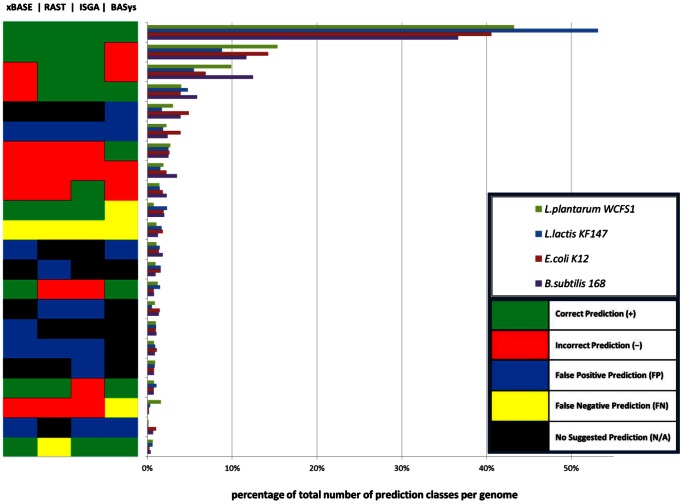
Variation in AGE predictions for four moderate GC% bacterial genomes. The start codon prediction accuracy by BASys, ISGA, RAST and xBASE is illustrated in this vertical bar-graph for four bacterial reference genomes: *B. subtilis* 168, *E. coli* K12 MG1655, *L. lactis* KF147 and *L. plantarum* WCFS1. On the y-axis, the different classes of predicted ORF starts compared to the respective reference genomes are shown. A black colored box is present only in combination with false positive predictions (blue). It signifies that for these mORFs no prediction data was provided by any of the other AGEs. A total of 82 unique color/prediction classes were defined. They were plotted on the y-axis and a number was assigned according to its prevalence per bacterial genome. These numbers are shown as a bar-graph on the x-axis: as a fraction/percentage of the total number of mORFs available for that genome. In order to reduce the number of classes, those that occurred on average less than 0.50% in the four genomes were removed leaving 22 prediction classes. See for all 82 classes Figure S2.

To assess the overlap in ORF start codon predictions by different AGEs we compared the predicted start codons to those in the original annotation for four moderate GC% reference genomes: *B. subtilis* 168, *E. coli* K12 MG1655, *L. lactis* KF147 and *L. plantarum* WCFS1 (Table 2). These four genomes are assumed to be a fair representation of moderate GC content genomes. Some AGEs better predict ORF start codons than other AGEs (Fig. S1), indicated by the different levels of correct- and incorrect predicted ORFs (e.g. ISGA consistently has the highest absolute number of correct predictions). This is also illustrated by the different combinations of ORF start codon prediction qualifications assigned for each AGE (e.g. correct-, incorrect-, false positive-, false negative- or N/A prediction), which were found for these four genomes (Fig. 4). For instance, more ORF start codons are correctly predicted by BASys compared to ISGA (2.6±0.1% and 1.7±0.4% respectively). Depending on the genome, either ISGA (83.6% correct predicted ORF start codons for *L. plantarum*) or RAST (82.7±2.3% correct predicted ORF start codons for *L. lactis*, *E. coli* and *B. subtilis*) performs best in respect to ORF start codon prediction accuracy (Fig. 4 and Fig. S1). Based on the four genomes, about half of the ORF start codons were correctly predicted by all four AGEs. The four AGEs perform inconsistent (Fig. 4) for a considerable fraction of the predicted ORFs (ranging from 40.6% for *L. lactis* KF147 to 57.9% for *B. subtilis* 168). In conclusion, for these four reference genomes, not one AGE will provide the best possible result for all ORFs (Fig. 4). We therefore hypothesize that a combination of AGEs might allow for more accurate prediction of start codon coordinates.

### Start Codon Prediction Performance by Majority Voting

**Figure 5:**
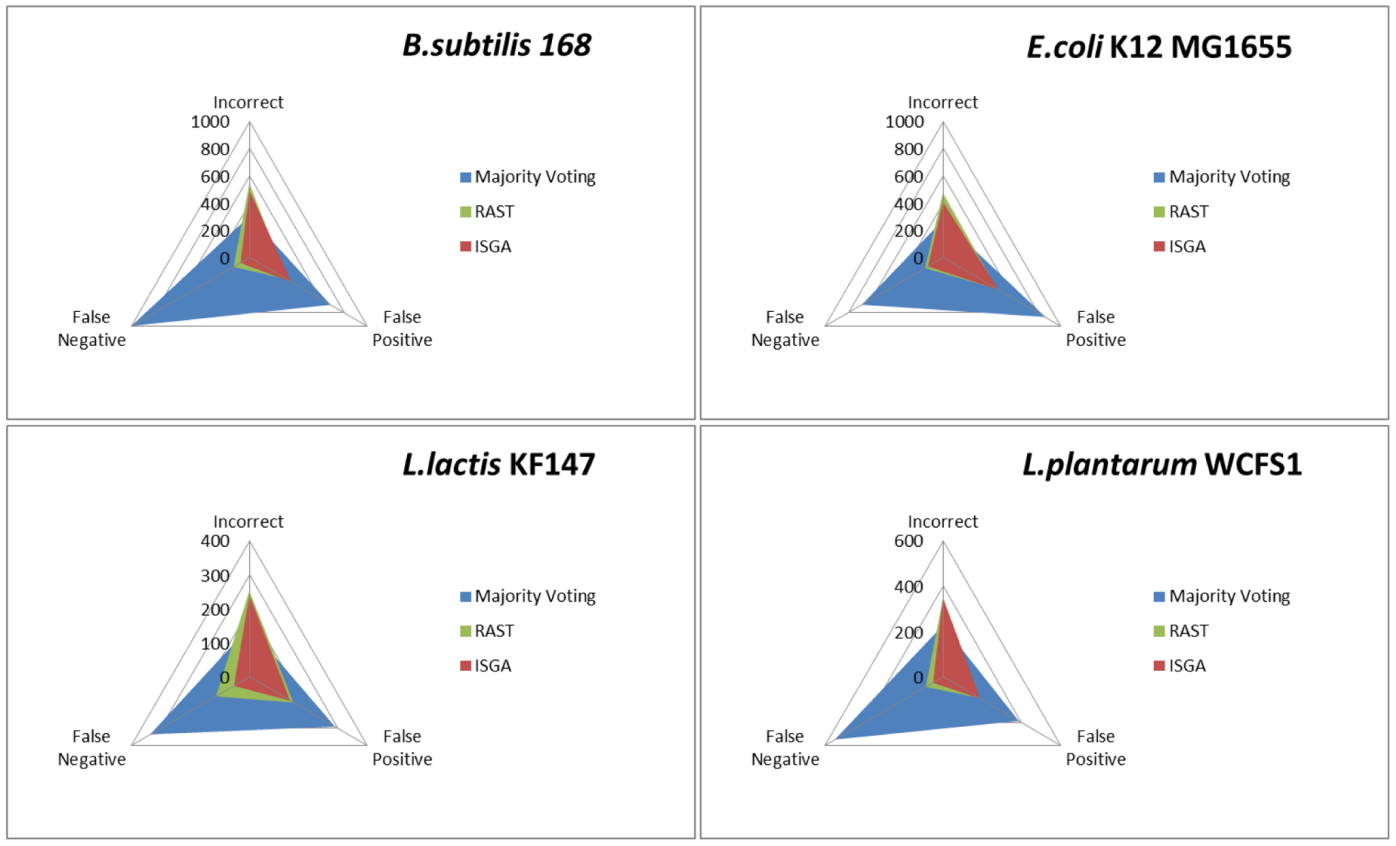
Start codon prediction performance by majority voting versus ISGA and RAST. This radar-plot illustrates start codon annotation results for four moderate GC% reference genomes *B. subtilis* 168, *E. coli* K12 MG1655, *L. lactis* KF147 and *L. plantarum* WCFS1 determined by majority voting with BASys, ISGA, RAST and xBASE predictions, or by a single AGE. With majority voting a prediction is trusted if more than 50% of the AGEs predict exactly the same start codon coordinate for a given mORF. Predictions were evaluated with the reference genomes (Table 2). The axis gridlines in all directions are in steps of 100 or 200 ORFs; with incorrect predicted ORF start codons (0°), false positively (FP) and false negatively (FN) predicted ORF start codons (respectively 120° and 240°).

Majority voting allows combining the annotation predictions from different AGEs and potentially results in more reliable predictions [18]. With majority voting, a start codon for a given ORF is based on the fact that it was predicted by most AGEs. The more AGEs are in consensus, the higher the confidence in the majority-voted start codon for that particular ORF. We evaluated the performance of majority voting against the single AGE with the lowest percentage of incorrect start codon predictions (ISGA or RAST, depending on the genome) on the four genomes. Majority voting introduces many more false positive (a predicted ORF was not annotated in the reference genome) ORFs (153–397) and false negative (ORF present in the reference genome, but not predicted by AGEs) ORFs (284–924) compared to ISGA or RAST (Fig. 5). However, the absolute number of incorrect predictions is also slightly lower with majority voting compared to ISGA or RAST alone. Likely, certain AGEs introduce inconsistencies in the voting results and thereby prevent correct predictions from being selected, in disfavor of other engines. Because predictions are only considered if a majority is reached, majority voting could lead to fewer predicted ORFs: ORFs for which voting results in a draw are missed. In conclusion, different AGEs may predict different ORFs correctly. As majority voting results in many false positive and false negative predictions, we hypothesize that specific combinations of AGEs possibly achieve better ORF start codon predictions.

### Start Codon Prediction Performance by Consensus Predictions

Another approach is to trust only the consensus prediction of (combinations of) AGEs. A prediction is only considered if all or a subset of engines predicts the same start codon. We therefore categorized predicted ORFs for which the start codon was determined in consensus for all combinations of two, three or four AGEs. Each of these determined consensus predictions was classified according to the reference genome in one of the following categories: (i) correct, (ii) incorrect, (iii) false negative or (iv) false positive (Fig. 2). Different combinations of two, three or four AGEs show different subsets of ORF start codons to be correctly predicted in *E. coli* K12 MG1655 (Figs. S3 and S4). For example, the combined consensus prediction of RAST and xBASE has a sensitivity (i.e. the coverage level with respect to the reference genome ORFs, taking into account both incorrectly and the correctly predicted ORFs; see *formula 3*) of 77.5% and a specificity (i.e. the percentage correct of predictions; see *formula 2*) of 86.0%, while the combination of RAST and BASys has a sensitivity of 58.3% and a specificity of 90.8%. Expectedly, the consensus prediction results of BASys, RAST and xBASE has a decreased sensitivity to 52.4%, but an increased specificity to 92.2%. Apparently, combining multiple AGEs allows predicting fewer ORF starts (lower sensitivity), but allows a more reliable start codon prediction (increased specificity). The combination of all four AGEs has the lowest sensitivity (50.1%) and the highest specificity (94.6%) for the *E. coli* K12 MG1655 data set. Similar observations were made for the other three genomes (Fig. S3): a sensitivity and specificity range of respectively 44.6% and 94.1% for *B. subtilis* 168, 50.5% and 96.5% for *L. lactis* KF147, 49.4% and 96.2% for *L. plantarum* WCFS1 was observed when this consensus prediction of all four AGEs was used.

Interestingly, some combinations of two AGEs provide a higher specificity compared to a combination of three engines; e.g. ISGA, RAST and xBASE (88.9%) versus BASys and ISGA (92.0%) or BASys and RAST (90.8%) (Fig. S3); this trend is observed for *E. coli* K12 MG1655 as well as for the other three genomes (Fig. S3). This leads to the postulate that serially applying consensus predictions of specific combinations of AGEs in order of specificity would allow better overall prediction compared to the alternatives: majority voting, the most reliable AGE only, or consensus predictions based on one AGE combination. As described above, approximately 50% of the ORF start codons can be correctly predicted by using the consensus of four AGEs. The question remains: how to reliably as possible predict the remaining half of ORF start codons?

### A Specific Path in Consensus Predictions of Different Combinations of Engines

**Figure 6:**
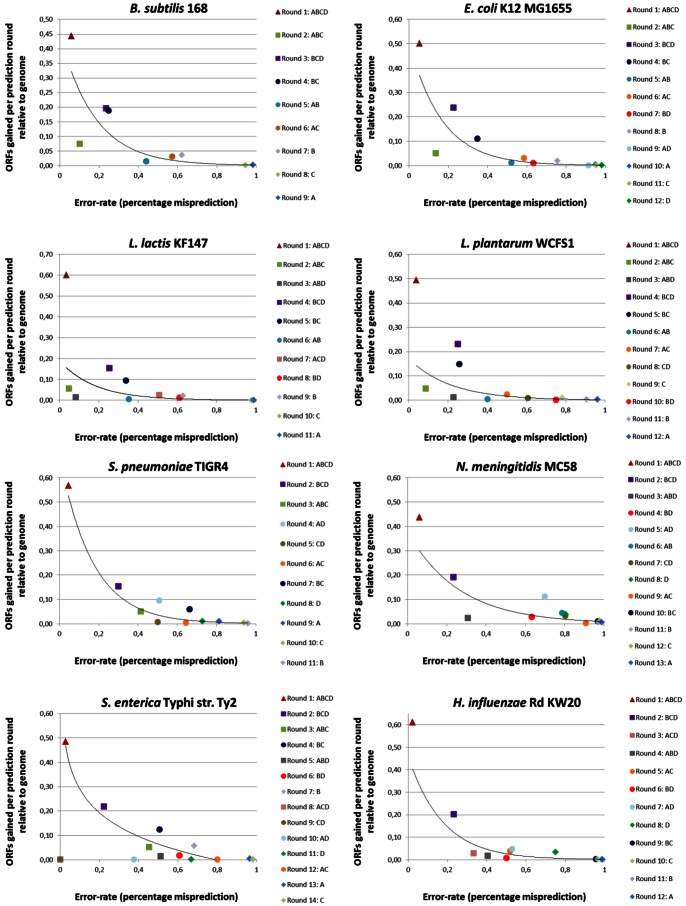
Applying multiple rounds of consensus predictions. Plotted in these graphs for four moderate GC% prokaryotic reference strains *B. subtilis* 168, *E. coli* K12 MG1655, *L. lactis* KF147 and *L. plantarum* WCFS1 (Table 2) is the error-rate for start codon coordinate prediction (x-axis) versus the new ORFs gained per prediction round (y-axis) for a selected AGE (diamond) or a selected combination of AGEs - two (circle), three (square) or four engines (triangle) - with the lowest calculated error-rate for that concerning round of prediction. ORFs were only taken into account when they were in consensus for their start codon coordinate prediction. Error-rates were calculated as discussed in materials and methods. A: BASys; B: ISGA; C: RAST and D: xBASE. Note that the trend line is merely for illustrative purposes: it does not signify an actual relation between the data points.

In the previous paragraphs it was shown that majority voting has a high false positive rate (Fig. 5). For this reason we are looking for alternative ways to maximize the prediction specificity. Therefore, our objective is to achieve a best-as-possible specificity for start codon prediction based on the twelve genomes. Unfortunately, in order to obtain high specificity we have to accept lower sensitivity, resulting in a lower (re)coverage of ORFs (see also Fig. S3). The consensus prediction of all four AGEs (Figs. S3 and S4) allows accurately predicting half of the actual ORF start codons present in the selected four moderate GC% genomes. If we also want to successfully predict the remaining ORFs with high specificity, they could be predicted with the consensus prediction of different AGE combinations (i.e. using consensus predictions from combinations of two or three AGEs). This process could be done via an iterative algorithm to arrive at an optimal order of consensus predictions. This path of serially applying combinations of AGEs ensures high specificity for prediction of ORF start codons for a genome of interest. The consensus-path method is explained further in Figure 3 and the materials and methods. From now on we refer to this optimal order of consensus predictions as consensus-path. From the numbers of correct-, incorrect, and false positive predictions (Figs. S1 and S2; and Fig. 4), we determined for each AGE and combinations of AGEs (see above) for the twelve reference genomes a sensitivity and error-rate (specificity) in ORF start codon prediction (Fig. S3). These error-rates allowed determining for each round of the path an error-rate for each AGE or combination of AGEs (Fig. 3). After each round, the AGE or a combination of AGEs was selected with the highest specificity (Fig. 3). The order of AGE combinations resulting in the highest specificity was quite similar in all eight moderate GC% bacterial genomes (Fig. 6) but less similar for the more extreme GC% genomes (Fig. S5).

The consensus-path consisted of using the ORF predictions of five specific combinations of AGEs applied consecutively (Figs. 3 and 6). We limited the number of successive rounds to five as relatively few new ORFs were added in additional rounds (Fig. 6). For instance round 6 added only 152 new ORFs for *E. coli* K12 MG1655 (Fig. 6). Notably, the specificity decreases with each round making the ORF start codon predictions of additional rounds inaccurate (e.g. a 58.6% error-rate for each prediction after round five in *E. coli* K12 MG1655, strongly increasing for the successive rounds) (Fig. 6). Our consensus-path approach enables one to estimate the reliability of prediction, and thus to assess the need for a start codon to be manually curated. An optimal order may work best for a given genome, but it might not work best for another genome. Therefore, we tested if a “conserved” consensus-path was present in the eight moderate GC% genomes (Figs. S6A and S6B).

**Figure 7:**
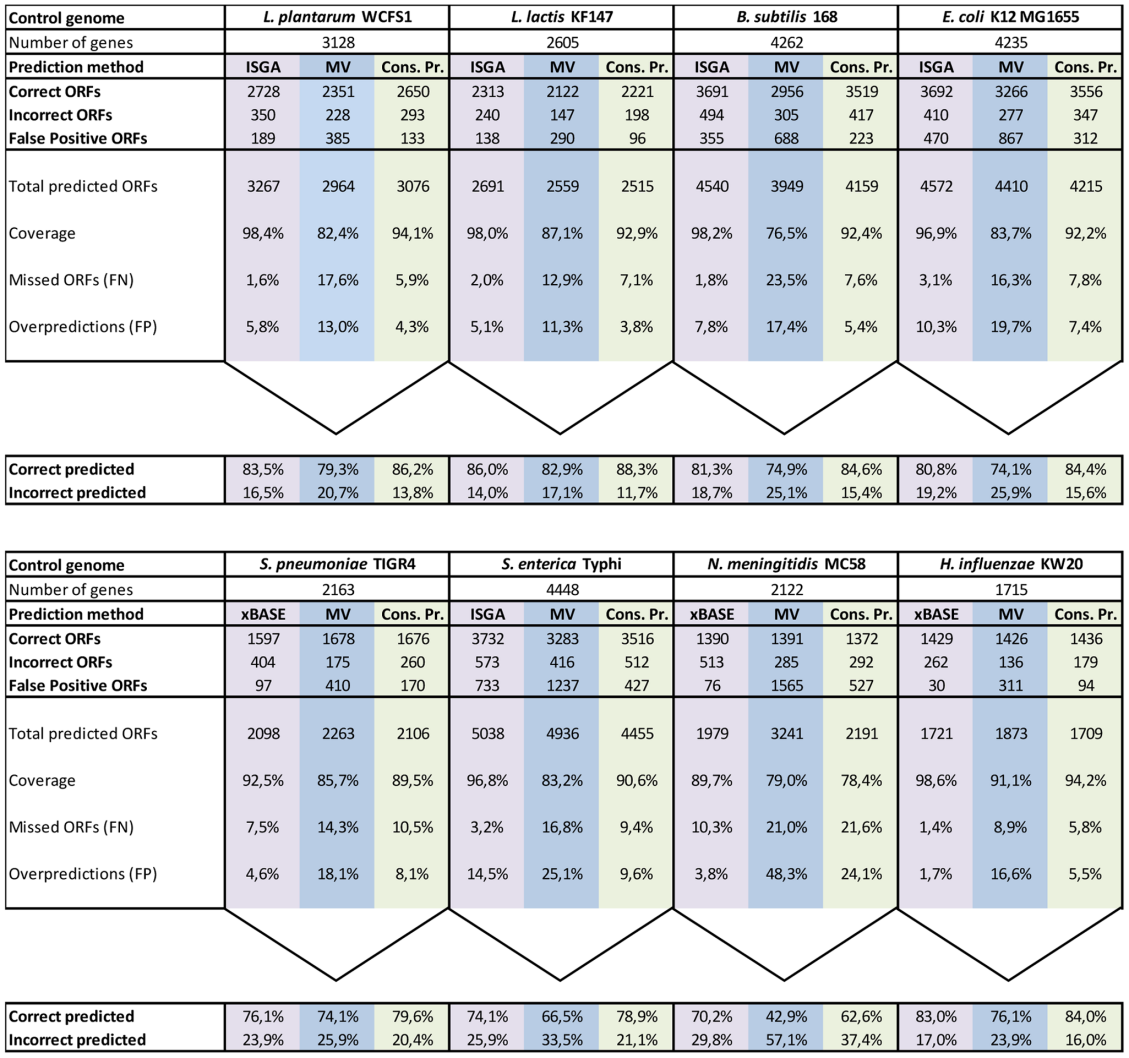
Analysis of start codon prediction performance for single engines, majority voting and consensus prediction. For three methods of processing AGE prediction data: (i) the single, most reliable AGE with highest impact (ISGA or xBASE, this varies between test strains; calculated with *formula 4*), (ii) majority voting (MV) and (iii) consensus predictions (Cons. Pr.) are shown for four moderate GC% reference strains from different species: *B. subtilis* 168, *E. coli* K12 MG1655, *L. lactis* KF147 and *L. plantarum* WCFS1. The number of mORFs is indicated which were incorrect-, correct- or false positive predictions according to the reference genomes, by applying each method of prediction. With majority voting a prediction is trusted if more than 50% of the AGEs for a mORF predict exactly the same start codon coordinate for that ORF. With consensus predictions we trust only the in consensus start codon prediction by a combination of AGEs. Also shown are: coverage (percentage correctly and incorrectly predicted ORFs, which are present in the reference genome), the total number of predicted ORFs, the fraction of missed ORFs according to the reference genome (FN; false negatives), and the fraction of over-predicted ORFs (FP; false positives). Bottom row (below the arrow heads): the predicted ORFs and the percentage of correct and incorrect predictions for the three methods.

For the eight moderate GC% genomes the paths appear to be similar (Figs. S6A and S6B). To determine the consensus-path that is most conserved across the eight genomes, we ranked for each genome the AGE combinations based on specificity (Figs. S6A and S6B). A simple formula (*formula 4*) was applied. It takes into account the rank, specificity and sensitivity to determine for that AGE (combination) an impact on correctly predicting ORF start codons (Fig. 6; see also Fig. S5 for the extreme GC% genomes). The five selected AGE combinations were: BASys-ISGA-RAST-xBASE in the first round, ISGA-RAST-xBASE in the second, BASys-ISGA-RAST in the third, ISGA-RAST in the fourth and BASys-RAST-xBASE in the fifth round. This path was applied to the eight reference genomes (Fig. 7). This resulted in a 9.7±4.4% gain in precision compared to majority voting and a 1.7±3.7% gain in precision compared to the single best performing AGE (Fig. 7). Based on the specificity of particular AGE combinations in the five rounds we derive a so-called projected confidence value. It is calculated as the average specificity of a particular AGE combination over the eight genomes (Fig. 6; calculated with *formula 2*). The overall projected confidence values (i.e. probability of an ORF start codon prediction to be correct) for these AGE combinations were calculated to be: 95.7±1.4% for a consensus of BASys-ISGA-RAST-xBASE, 75.6±2.3% for ISGA-RAST-xBASE, 67.6±26.0% for BASys-ISGA-RAST, 53.1±19.1% for ISGA-RAST and 42.6±12.3% for BASys-RAST-xBASE.

### Consistently Mis-predicted ORFs

Applying any AGE results in ORFs that are either not predicted or incorrectly predicted compared to the reference ORFs. For the four moderate GC% genomes *E. coli* K12 MG1655, *B. subtilis* 168, *L. plantarum* WCFS1, *L. lactis* KF147 we investigated whether these ORFs share common characteristics. Many of the ORFs present in the reference genomes were consistently mis-predicted (i.e. ORF is missed or has an incorrectly predicted start codon) by the AGEs: 208 for *E. coli*, 237 for *B. subtilis*, 103 for *L. plantarum* and 93 for *L. lactis* (Fig. 4; Figs. S1 and S2). If we would be able to understand why these predictions are going wrong, it might be possible to improve current AGEs. We therefore analyzed false negative ORFs that were consistently missed by all four AGEs (93 for *E. coli*, 62 for *B. subtilis*, 37 for *L. plantarum* and 49 for *L. lactis*) and the ORFS with consistently incorrectly predicted start codons by all four AGEs (115 for *E. coli*, 175 for *B. subtilis*, 66 for *L. plantarum* and 44 for *L. lactis*), to determine whether they share characteristics that might explain their incorrect start codon prediction. Smaller reference ORFs (<750 nucleotides; nt) are significantly more often missed by all AGEs (false negatives) (Fig. S7; chi-square p-values: <0.0001 for any of these four genomes). This was not the case for ORFs with consistently incorrectly start codons (Fig. S7; <750 nt chi-square p-values: *E. coli*: 0.70; *B. subtilis*: 0.07; *L. plantarum*: 0.48; *L. lactis*: 0.36). Possibly, AGEs have a threshold at a specific ORF length which could explain false negative ORF predictions.

The same phenomenon is observed for ORFs missed by our consensus-path prediction (i.e. ORFs not called after five rounds of consensus-paths). Compared to the corresponding reference genomes, smaller ORFs (i.e. <750 nt) are significantly more often missed (chi-square p-values <0.0001 for any of these four genomes).

Apart from ORF length, we tested these missed and incorrectly predicted ORFs for overrepresentation in other functional data (predictions) such as protein functionality (COG [25], DAVID database [26], [27], Pfam [13]), and subcellular protein localization (PSORTdb [28]). However, we did not find any significant overrepresentation of these functional annotations in the subset of incorrect predicted ORF start codons, nor in the false negative- and/or positive ORF subset (data not shown).

## Discussion

In order to improve start codon prediction in bacterial genomes by current AGEs we present an alternative method to majority voting [18]. We present a consensus-path for the prediction of bacterial ORFs by combining AGEs that could save researchers time, as manual curation of ORF start codons is tedious. As specificity in ORF start prediction is leading in ORF start curation the path is largely based on the specificity of AGEs (combinations). For eight moderate GC% genomes, we observe similar paths allowing us to postulate a generalized consensus-path for moderate GC% genomes. This consensus-path is specific for the four AGEs under study and might change as a result of changes within the ORF prediction procedures used in the AGEs and certainly when other AGEs are considered. Nevertheless, the application of an optimal path allows gaining sensitivity while maintaining a high specificity in ORF start codon prediction. In our case study, the consensus-path prediction is performed by assessing the consensus predictions of serially applying five AGE combinations: (i) BASys-ISGA-RAST-xBASE, (ii) ISGA-RAST-xBASE, (iii) BASys-ISGA-RAST, (iv) ISGA-RAST and (v) BASys-RAST-xBASE. Compared to majority voting we observe with our consensus path method an increase of 9.7±4.4% of predicted ORF start codons for which no further manual curation would be required. This equals easily hundreds of genes. Because application of consensus prediction is straight-forward, a researcher could without complex procedures benefit from an increase in annotation quality. Importantly, based on a novel projected confidence value one can determine ORFs for which likely an incorrect start codon prediction has been made. These ORFs can subsequently be targeted for manual curation. In addition, this information could be used to improve current ORF start codon prediction services and tools. Especially ORFs acquired in the fifth round of consensus-path prediction (*E. coli*: 99; *B. subtilis*: 97; *L. plantarum*: 58; *L. lactis*: 63) are notoriously difficult to predict and likely require manual curation.

The results presented in this case study have been achieved with the ORF start codon predictions of web-based genome annotation services that are free to use. Although our conclusions are based on four bacterial AGEs (Table 1) and eight moderate GC% bacterial genomes, we believe that this consensus-path based on the four AGEs can be applied to other moderate GC% organisms. Moreover, the concept of our consensus-path can be applied to other AGEs and other moderate GC% genomes. Compared to majority voting, the consensus prediction of multiple AGEs over multiple rounds of prediction results in 9.7±4.4% more correct predictions and 12.7±4.8% less false positive and 6.9±0.5% less false negative predictions (Fig. 7). Compared to the single, most reliable engines with the highest impact (either ISGA or xBASE) there is a slight gain in correct start codon prediction with 1.7±3.7% more correct predictions. However, 1.8±2.3% more false positives and 5.6±1.8% more false negatives were observed because some ORFs are over- or under predicted by a combination of AGEs but not by another single AGE. In any case, after applying the consensus-path approach, one is still able to supplement the already acquired predictions with those of a single AGE. However, these added predictions will generally be more error-prone and no high level of projected confidence can be assigned to them.

In the coming years, automated (genome) annotation processes will keep continuing to improve to the point that, ideally, barely any manual curation will be necessary. Currently, however, researchers have to be careful with interpreting AGE ORF start predictions. From this study we conclude that every AGE has its own unique strengths and weaknesses, likely related to the underlying tools and protocols used (Table S1). Therefore, it could be rewarding to combine AGEs in order to benefit from comparative annotation strategies; and thus to further increase the specificity and sensitivity of ORF predictions for a given genome.

## Materials and Methods

### Genome Sequences and Annotations

The predicted ORF start codon coordinates were evaluated by validation with ORFs from reference genomes from twelve well-studied strains of different bacterial species. This set of twelve genomes consists of eight moderate GC% genomes: *E. coli* K12 MG1655 (50% GC) [29], *B. subtilis* 168 (44%) [30], *L. plantarum* WCFS1 (44%) [31], *L. lactis* KF147 (35%) [32], *S. pneumoniae* TIGR4 (40%) [33], *S. enterica* subsp. *enterica* serovar Typhi str. Ty2 (52%) [34], *N. meningitidis* MC58 (52%) [35] and *H. influenzae* Rd KW20 (38%) [36]. In addition, four more extreme GC% genomes were analyzed: *M. tuberculosis* H37rv (66% GC) [37], *M. mobile* 163K (25%) [38], *P. putida* KT2440 (61%) [39] and *S. coelicolor* A3(2) (72%) [40] (Table 2).

### AGEs

For this study four microbial AGEs were selected: BASys [41], ISGA [42], RAST [43] and xBASE [44], [45] (Table 1). Although there are other excellent online AGEs available, we selected these four based on their relatively short queues and processing time, and on their easily exportable annotation data. These engines are all online, free-of-charge initiatives for non-profitable scientific research. FASTA-files of the reference genomes (Table 2) were uploaded to the engines, and default server settings were applied with exception of the following non-trivial options: for RAST: “automatically fix errors”, “fix frameshifts” and “backfill gaps” were selected; for ISGA: the standard/suggested “ISGA Prokaryotic Annotation” pipeline of February 2011 was employed. ISGA and xBASE use GLIMMER version 3 for its ORF prediction, BASys uses GLIMMER version 2.1.3. RAST uses a custom ORF caller called “RAST” (which was used in this study); however, RAST also allows the use of GLIMMER version 3.

### Comparison of ORF-predictions by Defining Meta ORFs

For comparing start codon coordinate positions we used in-house Perl scripts for analyzing AGE annotation data. This software package can be downloaded from https://trac.nbic.nl/companion/and is open for public use. This tool – which we have named COMPANION: an acronym for comparative genome annotation – evaluates the start- and stop-codon coordinates of (i) ORFs predicted by the different AGEs (Table 1), and (ii) ORFs provided by the reference genome (Table 2). It then groups these ORF coordinates into equivalent ORFs based on position and length of these ORFs. We name these ORF representatives meta ORFs (mORF). ORFs of which the start or stop codon differs less than 10% (of the ORF length of the shortest ORF of the two ORFs being compared) compared to its reference ORF start or stop codon were grouped into the same mORF prediction (Fig. 1). Perl scripts were used to extract from this table for each mORF the start codon for the respective engines. As both predictions and reference genome annotations are merged, for each mORF the predictions can be compared to the (if available) high quality reference ORF start codon annotations. The COMPANION tool was also used for statistical analysis and for determination of a consensus-path as mentioned below.

### Statistical Analysis on Meta ORFs

For each reference genome, the predicted start codons from the various AGEs were matched to those of the corresponding ORFs in the reference genome (Table 2 and Fig. 2). If a specific ORF from the reference genome was not represented by an ORF predicted by an AGE (combination), we defined that ORF start codon prediction to be false negative for those AGEs. If an ORF was predicted by an AGE but it was not present in the reference genome, we defined that ORF as a false positive prediction. When a predicted ORF start codon matched that in the reference genome (hence, start codon predictions from the same mORF; Fig. 1), it was considered a correct en therefore true positive prediction. In case a predicted ORF was present in a reference genome (same mORF) but its start codon did not match that of the reference genome, it was considered an incorrect prediction.

Error-rate was calculated as follows (*formula 1*):
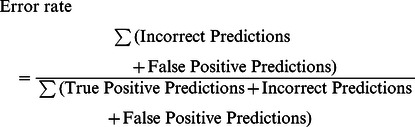



Specificity (a number between 0 and 1) was subsequently calculated (*formula 2*):
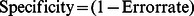



Sensitivity was calculated as follows (*formula 3*):
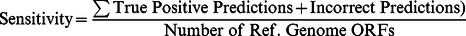



### Determination of the Consensus-path

Combining specificity, sensitivity and rank (where rank is the order in which AGE (combinations) are predicted; see Fig. 6 and Fig. S5), enables determining the impact of a certain prediction method (i.e. a single AGE, majority voting or a consensus-path) on the correct prediction of subset of ORF start codons under study. To account for AGE (combinations) that perform less on specific genomes, we incorporated the rank of prediction specificity into our formula for determining the *impact* for a prediction method. This was to account for that the order of prediction by an AGE (combination) is crucial in our approach (Fig. 3B). Based on the impact of a particular AGE (combination) for a particular genome, we can calculate the *average impact* over eight moderate GC% genomes of that AGE (combination). This impact for a prediction method was calculated as follows (*formula 4*):
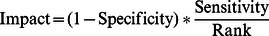



Formula 4 enables us to - for each genome - determine an impact value for each AGE (combination). This allows establishing a *general consensus path* (Fig. 3) by taking the highest impact values for AGE (combinations) over the selected eight moderate GC% genomes (Figs. S6A and S6B). This results in the average impact value.

Because we analyze genomes with trusted ORF start annotations (Table 2), we can derive *projected confidence values* for the selected AGE (combinations) part of the consensus-path. These are estimations of the probability of making correct ORF start codon predictions (*formula 2*) when applying certain AGE (combinations) to a new genome. Therefore, we are able to assign to each AGE (combination) its own *general projected confidence value*, which is an average of all projected confidence values for an AGE (combination) over the eight moderate GC% genomes.

## Supporting Information

Figure S1 **Variation in AGE ORF start- and stop codon predictions for four moderate GC% bacterial genomes.**
(PDF)

Figure S2 **Variation in consensus AGE ORF start codon predictions for four moderate GC% bacterial genomes.**
(PDF)

Figure S3 **AGE annotation prediction specificity and ORF recovery.**
(PDF)

Figure S4 **Percentage ORFs incorrect when AGEs have a consensus start codon coordinate prediction.**
(PDF)

Figure S5 **Applying multiple rounds of consensus prediction for four more extreme GC% genomes.**
(PDF)

Figure S6 **Selecting the most optimal consensus-path.**
(PDF)

Figure S7 **ORF length bias in false negatively predicted ORFs.**
(PDF)

Table S1 **Key functionalities in various AGEs.**
(PDF)
