# Supplementary material for: Reduce Manual Curation by Combining Gene Predictions from Multiple Annotation Engines, a Case Study of Start Codon Prediction
Source: PLoS One. 2013 May 10;8(5):e63523. doi: 10.1371/journal.pone.0063523 (PMC3651085; doi:10.1371/journal.pone.0063523)
Supplement: Figure S1 — Variation in AGE ORF start- and stop codon predictions for four moderate GC% bacterial genomes. (PDF) [file pone.0063523.s001.pdf]

Figure S1. Variation in AGE ORF start- and stop codon predictions for four moderate GC% bacterial genomes.

Represented in bar-graphs are the numbers of total, correct, incorrect, false positive (FP) and false negative (FN) ORF start codon (**Fig. S1A**) and ORF stop codon (**Fig. S1B**) predictions by the BASys, ISGA, RAST and xBASE AGEs (Table 1) in *B. subtilis* 168, *E. coli* K12 MG1655, *L. lactis* KF147 and *L. plantarum* WCFS1 reference genomes (Table 2). FP- and FN predictions were not defined as incorrect predictions, to distinguish between the various types of mis-predictions. In addition, for each reference genome (Ref. Genome), the total number of ORFs present in its genome according to the latest genome information is indicated.

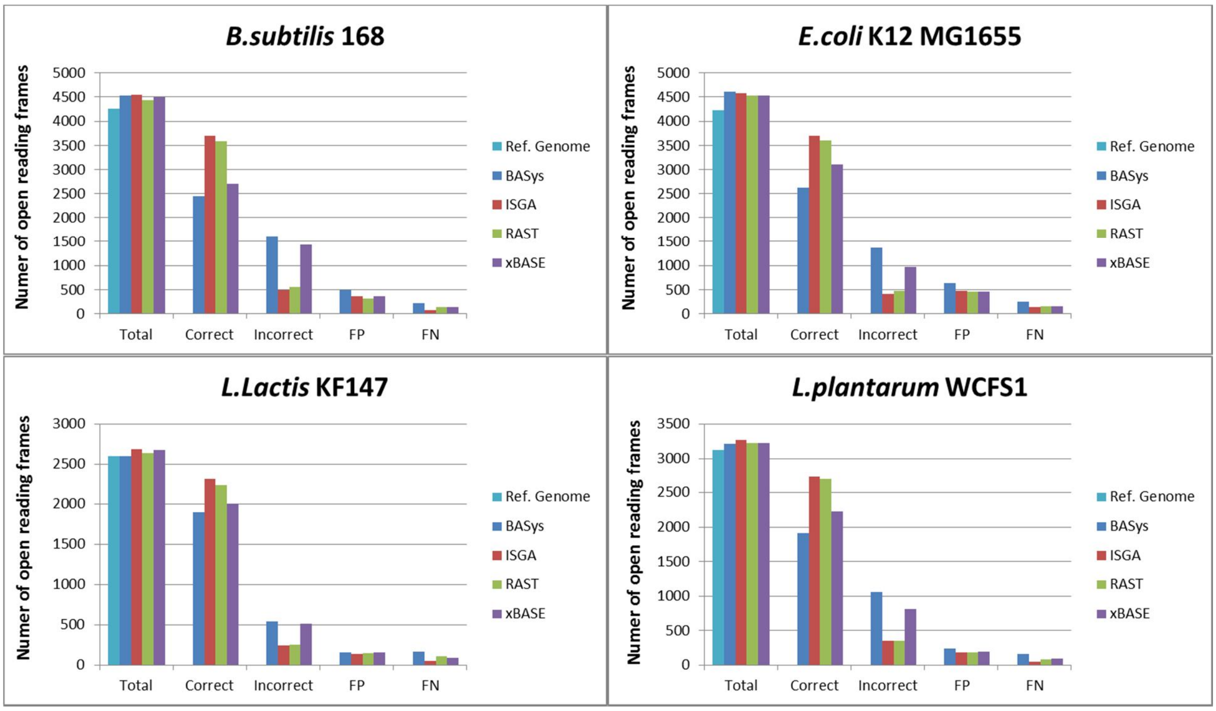

Figure S1A

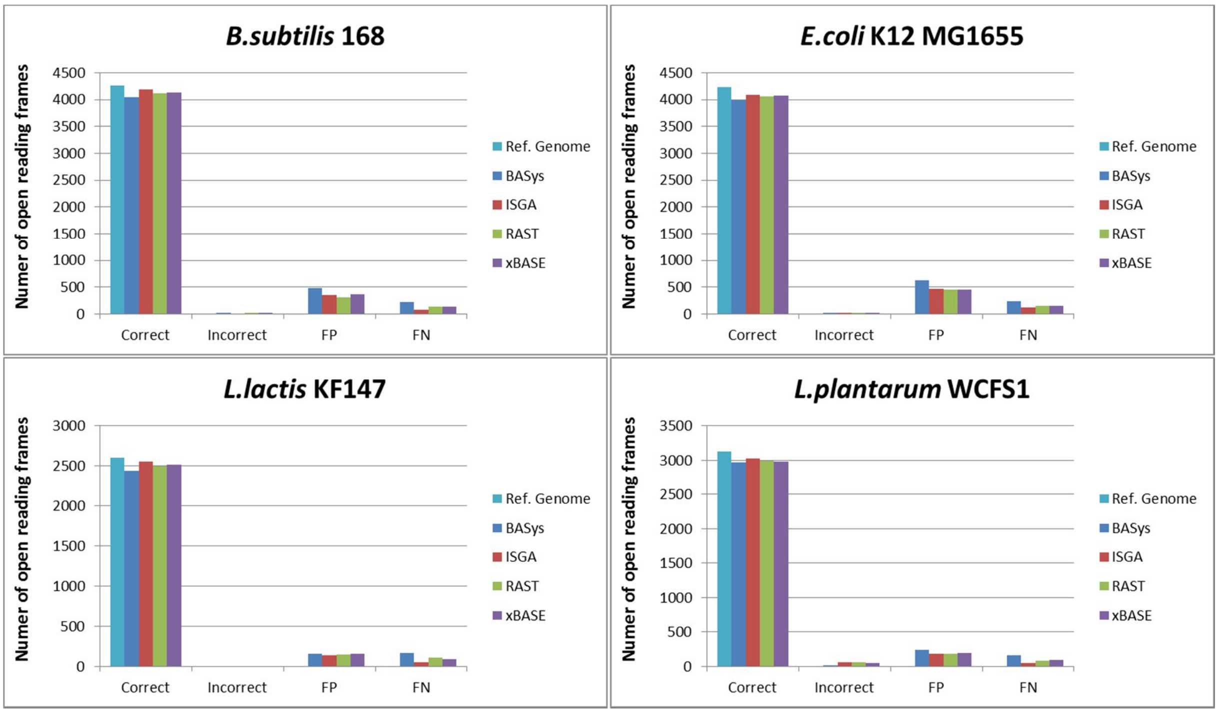
B.subtilis 168
E.coli K12 MG1655
L.lactis KF147
L.plantarum WCFS1
Numer of open reading frames
Correct
Incorrect
FP
FN
Ref. Genome
BASys
ISGA
RAST
xBASE

Figure S1B
