# Supplementary material for: Reduce Manual Curation by Combining Gene Predictions from Multiple Annotation Engines, a Case Study of Start Codon Prediction
Source: PLoS One. 2013 May 10;8(5):e63523. doi: 10.1371/journal.pone.0063523 (PMC3651085; doi:10.1371/journal.pone.0063523)
Supplement: Figure S2 — Variation in consensus AGE ORF start codon predictions for four moderate GC% bacterial genomes. (PDF) [file pone.0063523.s002.pdf]

Figure S2. Variation in consensus AGE ORF start codon predictions for four moderate GC% bacterial genomes.

The start codon prediction accuracy by BASys, ISGA, RAST and xBASE is illustrated in this vertical bar-graph for four moderate GC content, but distant bacterial genomes: *B. subtilis* 168, *E. coli* K12 MG1655, *L. lactis* KF147 and *L. plantarum* WCFS1. On the y-axis, the different classes of predicted ORF starts compared to the respective reference genomes are shown. Blue: false positive predictions (FP); yellow: false negative predictions (FN); green: correct (+); and red: incorrect (-) predictions. A black colored box is present only in combination with false positive predictions (blue). It signifies that for these mORFs no prediction data was provided by any of the other AGEs. A total of 82 unique color/prediction classes were defined. They are plotted on the y-axis and a number was assigned according to its prevalence per bacterial genome. These numbers are shown as a bar-graph on the x-axis: as a fraction/percentage of the total number of mORFs available for that genome. Note that for visualization reasons the x-axis has been cut-off after 15%.

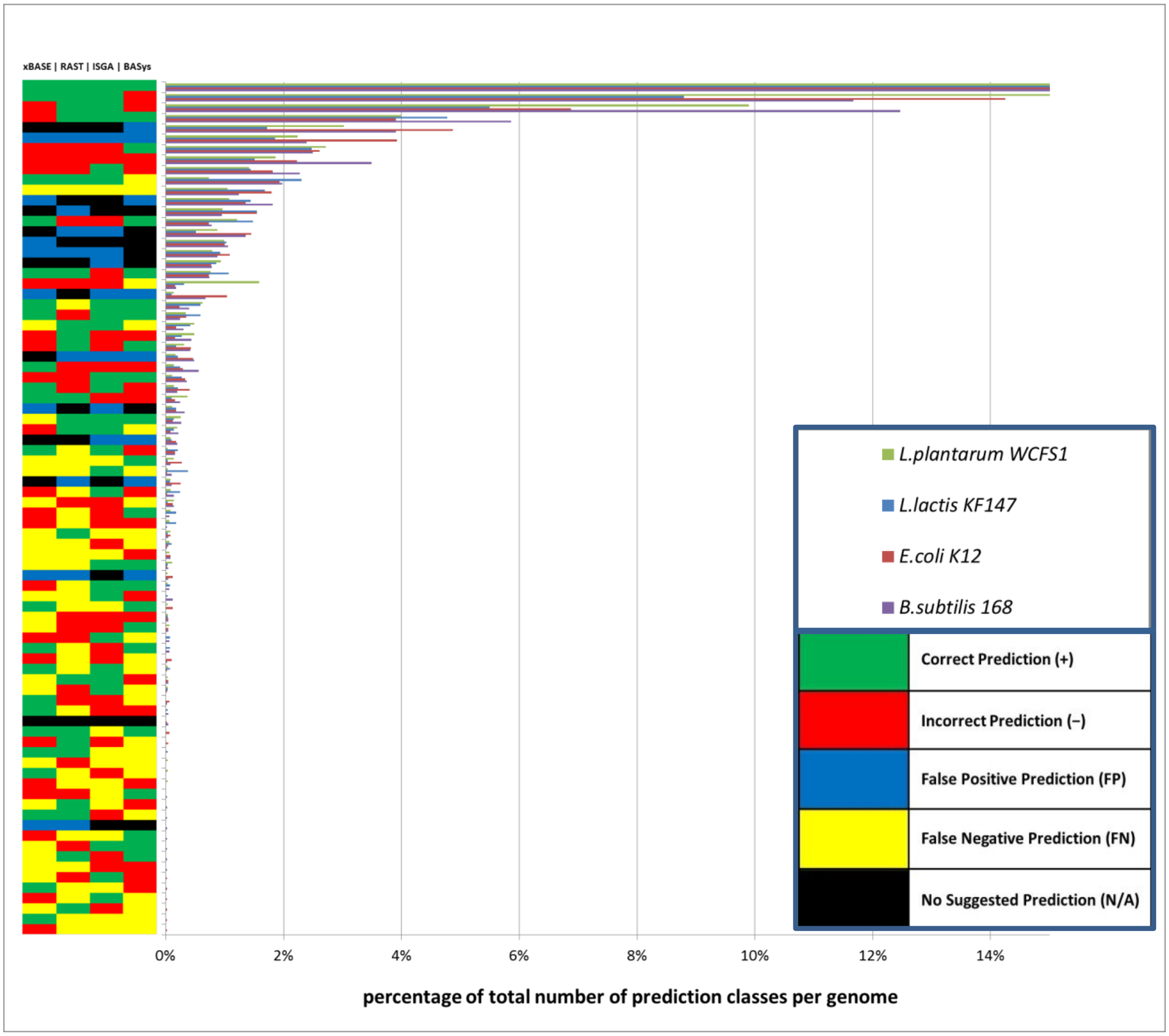
xBASE | RAST | ISGA | BASys
L.plantarum WCFS1
L.lactis KF147
E.coli K12
B.subtilis 168
Correct Prediction (+)
Incorrect Prediction (–)
False Positive Prediction (FP)
False Negative Prediction (FN)
No Suggested Prediction (N/A)
0%
2%
4%
6%
8%
10%
12%
14%
percentage of total number of prediction classes per genome
