# Supplementary material for: Reduce Manual Curation by Combining Gene Predictions from Multiple Annotation Engines, a Case Study of Start Codon Prediction
Source: PLoS One. 2013 May 10;8(5):e63523. doi: 10.1371/journal.pone.0063523 (PMC3651085; doi:10.1371/journal.pone.0063523)

Figure S3. AGE annotation prediction specificity and ORF recovery.

The specificity of ORF start codon prediction for four AGEs (diamond) and of various combinations of these AGEs is plotted for four reference annotations: for two (circle), three (square) or four engines (triangle). mORFs were only taken into account when they were in consensus for their start codon coordinate prediction. For specificity and sensitivity - or re(coverage) - calculations see materials and methods. A: BASys; B: ISGA; C: RAST and D: xBASE.

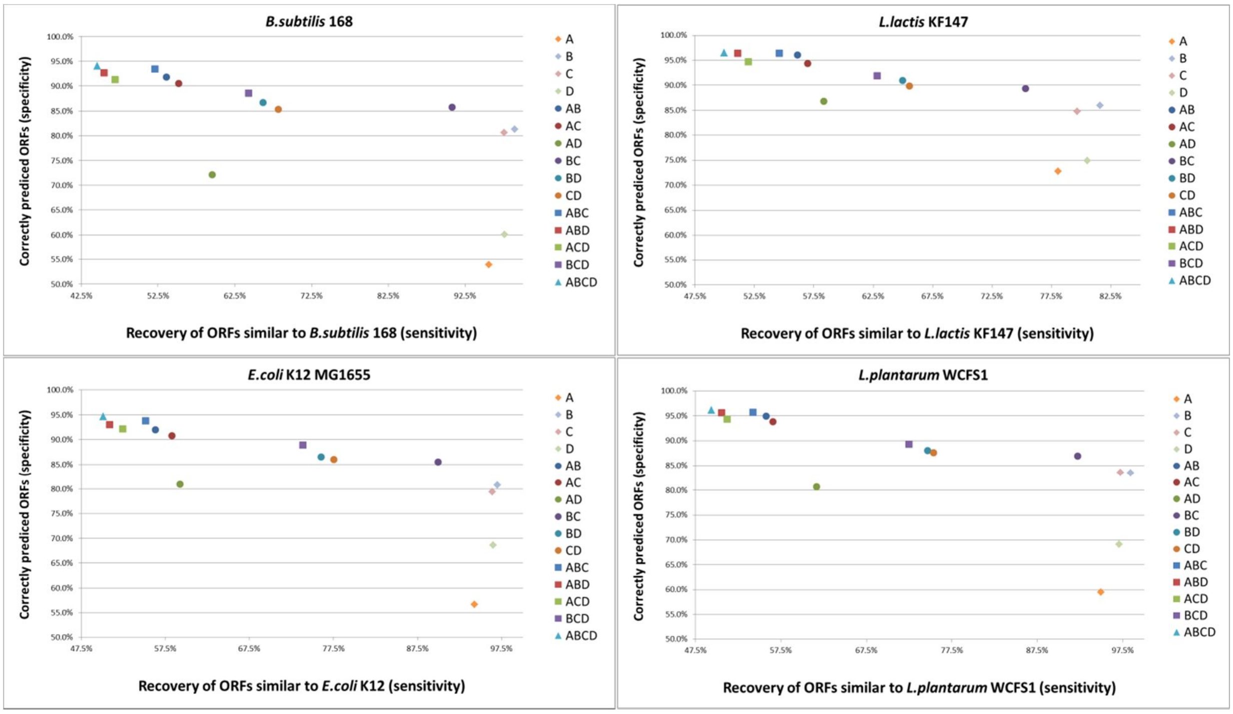

Supplement: Figure S3 — AGE annotation prediction specificity and ORF recovery. (PDF) [file pone.0063523.s003.pdf]
