# Supplementary material for: Reduce Manual Curation by Combining Gene Predictions from Multiple Annotation Engines, a Case Study of Start Codon Prediction
Source: PLoS One. 2013 May 10;8(5):e63523. doi: 10.1371/journal.pone.0063523 (PMC3651085; doi:10.1371/journal.pone.0063523)

Figure S4. Percentage ORFs incorrect when AGEs have a consensus start codon coordinate prediction.

The error-rates for consensus start codon coordinate prediction for four AGEs (Table 1) and of various combinations of these four AGEs are plotted for the four reference organisms. Error-rates were calculated as discussed in materials and methods. A: BASys; B: ISGA; C: RAST and D: xBASE.

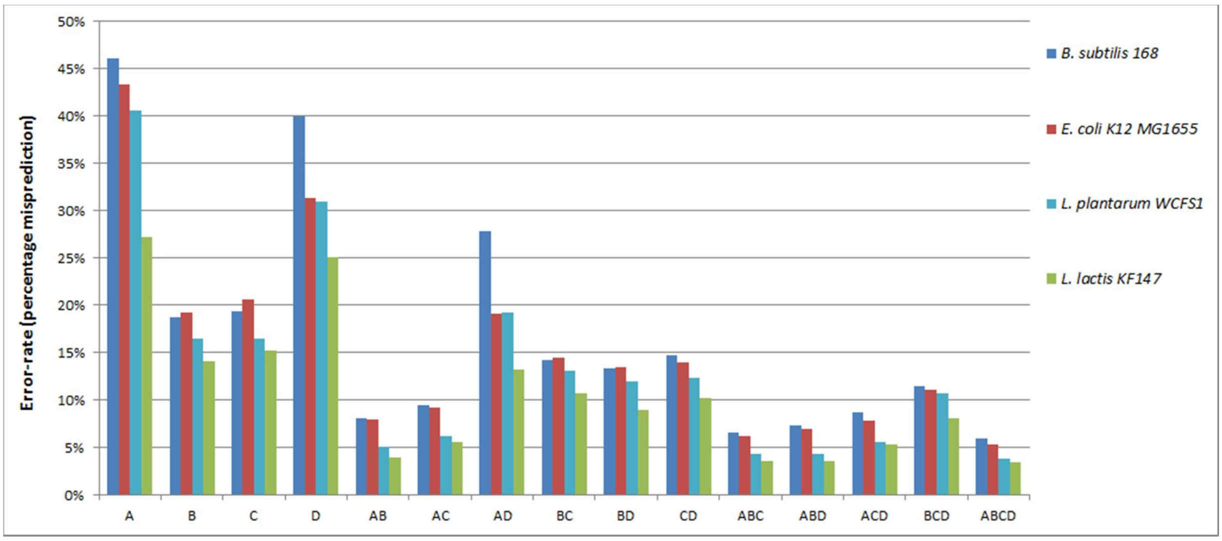

Supplement: Figure S4 — Percentage ORFs incorrect when AGEs have a consensus start codon coordinate prediction. (PDF) [file pone.0063523.s004.pdf]
