# Supplementary material for: Reduce Manual Curation by Combining Gene Predictions from Multiple Annotation Engines, a Case Study of Start Codon Prediction
Source: PLoS One. 2013 May 10;8(5):e63523. doi: 10.1371/journal.pone.0063523 (PMC3651085; doi:10.1371/journal.pone.0063523)

Figure S5. Applying multiple rounds of consensus prediction for four more extreme GC% genomes.

Error-rate (x-axis) for start codon coordinate prediction for a (combination of) AGE(s) versus new ORFs gained per prediction round (y-axis) for the four more extreme GC% genomes. Diamond: a particular AGE. Circle: 2 AGEs. Square: 3 AGEs. Triangle: 4 AGEs. AGE combinations chosen per round are based on that AGE combination with the lowest error-rate. ORFs were only taken into account when they were in consensus for their start codon coordinate prediction. Error-rates were calculated as discussed in materials and methods. A: BASys; B: ISGA; C: RAST and D: xBASE. Note that the trend line is merely for illustrative purposes: it does not signify an actual relation between the data points.

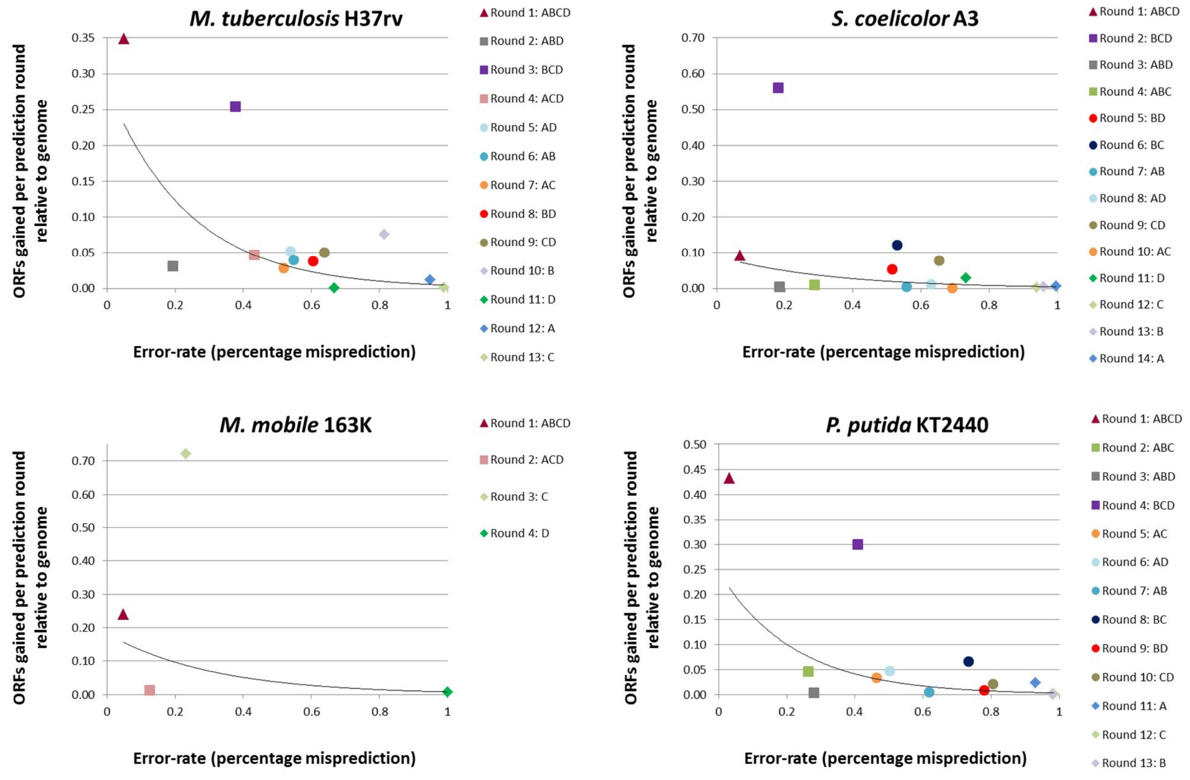

Supplement: Figure S5 — Applying multiple rounds of consensus prediction for four more extreme GC% genomes. (PDF) [file pone.0063523.s005.pdf]
