# Supplementary material for: Reduce Manual Curation by Combining Gene Predictions from Multiple Annotation Engines, a Case Study of Start Codon Prediction
Source: PLoS One. 2013 May 10;8(5):e63523. doi: 10.1371/journal.pone.0063523 (PMC3651085; doi:10.1371/journal.pone.0063523)
Supplement: Figure S6 — Selecting the most optimal consensus-path. (PDF) [file pone.0063523.s006.pdf]

Figure S6. Selecting the most optimal consensus-path.

The figures below illustrate and explain how after calculating an individual consensus-path for each genome (see Fig. 6) a more general consensus-path was formulated based on the paths of individual genomes. We show the paths based on the eight moderate- and four more extreme GC% genomes.

Figure S6A: We were able to calculate the impact of a certain AGE (combination) using *formula 4* (materials and methods) by taking into account: (1) sensitivity (i.e. the coverage, in other words, the percentage of ORFs predicted of the original known annotation), (2) specificity (i.e. the percentage of correctly predicted ORFs in the fraction of ORFs covered by the sensitivity), and (3) the rank or order in the consensus-path for a genome (as seen in Fig. 6). Dark gray blocks: a certain AGE (combination) was not part of the consensus-path for that genome (see Fig. 3 for these criteria). The average impact shown on the right is calculated by averaging the impact of all eight moderate GC% genomes (see *formula 4*). The table was sorted on the average impact. As a result, the most optimal general consensus-path could be formulated, as shown on the left. This path consists of BASys-ISGA-RAST-xBASE in the first round, ISGA-RAST-xBASE in the second, BASys-ISGA-RAST in the third, ISGA-RAST in the fourth and BASys-RAST-xBASE in the fifth round of annotation prediction comparison. Through this representation, the resemblance of each individual genome to the general consensus-path can also be visually assessed.

Figure S6B: A simplified representation of the consensus path across the different genomes**.** Each iteration (first column) by our consensus-path methodology (as described in Fig. 3) results in an AGE (combination) providing the highest specificity, which is listed for every iteration for each genomes. The consensus-path shown on the right is derived from the calculations shown in Figure S6A. The more

extreme GC% genomes show less concordance in consensus paths compared to the moderate GC% genomes. B. sub: *B. subtilis* 168, E. col: *E. coli* K12 MG1655, L. lac: *L. lactis* KF147, L. pla: *L. plantarum* WCFS1, S. pne: *S. pneumoniae* TIGR4, S. ent: *S. enterica* subsp. *enterica* serovar Typhi str. Ty2, N. men: *N. meningitidis* MC58, H. inf: *H. influenzae* Rd KW20, M. tub: *M. tuberculosis* H37rv, M. mob: *M. mobile* 163K, S. coe: *S. coelicolor* A3(2), P. put: *P. putida* KT2440. A: BASys; B: ISGA; C: RAST and D: xBASE.

Moderate GC% Species

Path	*B. subtilis* B168				*E. coli* K12				*L. lactis* KF147				*L. plantarum* WCFS1				Average
	Rank	Sens.	Spec.	Impact	Rank	Sens.	Spec.	Impact	Rank	Sens.	Spec.	Impact	Rank	Sens.	Spec.	Impact	Impact
ABCD	1	0.4427	0.0594	0.4164	1	0.5011	0.0537	0.4742	1	0.6000	0.0349	0.5791	1	0.4936	0.0384	0.4747	0.4949
BCD	3	0.1958	0.2349	0.0499	3	0.2374	0.2271	0.0612	4	0.1547	0.2523	0.0289	4	0.2311	0.2503	0.0433	0.0648
ABC	2	0.0746	0.1023	0.0335	2	0.0504	0.1369	0.0217	2	0.0557	0.0467	0.0265	2	0.0486	0.0854	0.0222	0.0189
BC	4	0.1880	0.2494	0.0353	4	0.1107	0.3480	0.0180	5	0.0940	0.3383	0.0124	5	0.1490	0.2560	0.0222	0.0139
ACD									7	0.0242	0.5079	0.0017					0.0045
AD					9	0.0014	0.9143	0.0000									0.0043
ABD									3	0.0134	0.0811	0.0041	3	0.0118	0.2273	0.0030	0.0033
AC	6	0.0303	0.5725	0.0022	6	0.0314	0.5855	0.0022					7	0.0233	0.5000	0.0017	0.0013
AB	5	0.0146	0.4400	0.0016	5	0.0116	0.5200	0.0011	6	0.0050	0.3529	0.0005	6	0.0038	0.4000	0.0004	0.0010
CD													8	0.0090	0.6071	0.0004	0.0010
BD					7	0.0092	0.6324	0.0005	8	0.0123	0.6111	0.0006	10	0.0019	0.7500	0.0000	0.0009
B	7	0.0364	0.6210	0.0020	8	0.0189	0.7564	0.0006	9	0.0207	0.6279	0.0009	11	0.0026	0.9070	0.0000	0.0009
D					12	0.0005	0.9815	0.0000									0.0005
C	8	0.0009	0.9444	0.0000	11	0.0009	0.9524	0.0000	10	0.0004	0.9792	0.0000	9	0.0105	0.7821	0.0003	0.0003
A	9	0.0021	0.9831	0.0000	10	0.0045	0.9507	0.0000	11	0.0004	0.9892	0.0000	12	0.0029	0.9610	0.0000	0.0000

Path	*S. pneumoniae* TIGR4				*S. enterica* Typhi				*N. meningitidis* MC58				*H. influenzae* Rd KW20				Average
	Rank	Sens.	Spec.	Impact	Rank	Sens.	Spec.	Impact	Rank	Sens.	Spec.	Impact	Rank	Sens.	Spec.	Impact	Impact
ABCD	1	0.5673	0.0455	0.5415	1	0.4852	0.0276	0.4718	1	0.4378	0.0617	0.4108	1	0.6117	0.0228	0.5977	0.4949
BCD	2	0.1540	0.2991	0.0540	2	0.2179	0.2231	0.0846	2	0.1913	0.2332	0.0734	2	0.2029	0.2314	0.0780	0.0648
ABC	3	0.0509	0.4138	0.0099	3	0.0522	0.4521	0.0095									0.0189
BC	7	0.0587	0.6615	0.0028	4	0.1246	0.5064	0.0154	10	0.0094	0.9685	0.0000	9	0.0029	0.9556	0.0000	0.0139
ACD					8	0.0007	0.0000	0.0001					3	0.0286	0.3333	0.0063	0.0045
AD	4	0.0948	0.5081	0.0117	10	0.0011	0.3750	0.0001	5	0.1126	0.6992	0.0068	7	0.0478	0.5294	0.0032	0.0043
ABD					5	0.0144	0.5120	0.0014	3	0.0231	0.3065	0.0053	4	0.0175	0.4048	0.0026	0.0033
AC	6	0.0042	0.6429	0.0002	12	0.0004	0.8000	0.0000	9	0.0028	0.9077	0.0000	5	0.0379	0.5179	0.0037	0.0013
AB									6	0.0443	0.7867	0.0016					0.0010
CD	5	0.0069	0.5000	0.0007	9	0.0002	0.0000	0.0000	7	0.0320	0.8028	0.0009					0.0010
BD					6	0.0171	0.6061	0.0011	4	0.0273	0.6324	0.0025	6	0.0070	0.5000	0.0006	0.0009
B	11	0.0009	0.9600	0.0000	7	0.0567	0.6828	0.0026	11	0.0052	0.9725	0.0000	11	0.0006	0.9841	0.0000	0.0009
D	8	0.0092	0.7273	0.0003	11	0.0011	0.6667	0.0000	8	0.0377	0.8049	0.0009	8	0.0338	0.7500	0.0011	0.0005
C	10	0.0046	0.9400	0.0000	14	0.0007	0.9819	0.0000	12	0.0113	0.9802	0.0000	10	0.0023	0.9639	0.0000	0.0003
A	9	0.0102	0.8136	0.0002	13	0.0036	0.9649	0.0000	13	0.0061	0.9884	0.0000	12	0.0012	0.9890	0.0000	0.0000

Extreme GC% Species

Path	*M. tuberculosis* H37rv				*M. mobile* 163K				*S. coelicolor* A3				*P. putida KT2440*				Average
	Rank	Sens.	Spec.	Impact	Rank	Sens.	Spec.	Impact	Rank	Sens.	Spec.	Impact	Rank	Sens.	Spec.	Impact	Impact
ABCD	1	0.3488	0.0494	0.3315	1	0.2390	0.0491	0.2273	1	0.0928	0.0687	0.0864	1	0.4322	0.0309	0.4189	0.4949
BCD	3	0.2542	0.3770	0.0528					2	0.5608	0.1808	0.2297	4	0.2993	0.4089	0.0442	0.0648
ABC									4	0.0091	0.2875	0.0016	2	0.0455	0.2623	0.0168	0.0189
BC									6	0.1215	0.5302	0.0095	8	0.0664	0.7343	0.0022	0.0139
ACD	4	0.0470	0.4316	0.0067	2	0.0121	0.1250	0.0053									0.0045
AD	5	0.0517	0.5381	0.0048					8	0.0121	0.6289	0.0006	6	0.0472	0.5019	0.0039	0.0043
ABD	2	0.0309	0.1938	0.0125					3	0.0046	0.1842	0.0012	3	0.0035	0.2800	0.0008	0.0033
AC	7	0.0285	0.5182	0.0020					10	0.0014	0.6923	0.0000	5	0.0336	0.4635	0.0036	0.0013
AB	6	0.0399	0.5473	0.0030					7	0.0038	0.5581	0.0002	7	0.0041	0.6182	0.0002	0.0010
CD	9	0.0507	0.6390	0.0020					9	0.0786	0.6532	0.0030	10	0.0208	0.8053	0.0004	0.0010
BD	8	0.0379	0.6051	0.0019					5	0.0529	0.5142	0.0051	9	0.0083	0.7800	0.0002	0.0009
B	10	0.0760	0.8151	0.0014					13	0.0038	0.9593	0.0000	13	0.0004	0.9800	0.0000	0.0009
D	11	0.0002	0.6667	0.0000	4	0.0061	1.0000	0.0000	11	0.0292	0.7319	0.0007					0.0005
C	13	0.0015	0.9896	0.0000	3	0.7201	0.2325	0.1842	12	0.0026	0.9394	0.0000	12	0.0007	0.9833	0.0000	0.0003
A	12	0.0116	0.9491	0.0000					14	0.0068	0.9961	0.0000	11	0.0232	0.9297	0.0001	0.0000

Figure S6A

	Moderate GC% Species									Extreme GC% Species			
Iteration	*B. sub..*	*E. col..*	*L. lac..*	*L. pla..*	*S. pne..*	*S. ent..*	*N. men..*	*H. inf..*		*M. tub..*	*M. mob..*	*S. coe..*	*P. put..*
1	ABCD	ABCD	ABCD	ABCD	ABCD	ABCD	ABCD	ABCD		ABCD	ABCD	ABCD	ABCD
2	ABC	ABC	ABC	ABC	BCD	BCD	BCD	BCD		ABD	ACD	BCD	ABC
3	BCD	BCD	ABD	ABD	ABC	ABC	ABD	ACD		BCD	C	ABD	ABD
4	BC	BC	BCD	BCD	AD	BC	BD	ABD		ACD	D	ABC	BCD
5	AB	AB	BC	BC	CD	ABD	AD	AC		AD		BD	AC
6	AC	AC	AB	AB	AC	BD	AB	BD		AB		BC	AD
7	B	BD	ACD	AC	BC	B	CD	AD		AC		AB	AB
8	C	B	BD	CD	D	ACD	D	D		BD		AD	BC
9	A	AD	B	C	A	CD	AC	BC		CD		CD	BD
10		A	C	BD	C	AD	BC	C		B		AC	CD
11		C	A	B	B	D	B	B		D		D	A
12		D		A		AC	C	A		A		C	C
13						A	A			C		B	B
14						C						A	D
GC%	44%	50%	35%	44%	40%	52%	52%	38%		66%	25%	75%	61%

Consensus Path:

1	ABCD
2	BCD
3	ABC
4	BC
5	ACD

Figure S6B
