# Supplementary material for: Reduce Manual Curation by Combining Gene Predictions from Multiple Annotation Engines, a Case Study of Start Codon Prediction
Source: PLoS One. 2013 May 10;8(5):e63523. doi: 10.1371/journal.pone.0063523 (PMC3651085; doi:10.1371/journal.pone.0063523)

Figure S7. ORF length bias in false negatively predicted ORFs.

Plotted in bar-graphs for moderate GC% species *B. subtilis* 168, *E. coli* K12 MG1655, *L. lactis* KF147 and *L. plantarum* WCFS1 is the relative number of ORFs present in the complete reference genome (purple), in the subset of incorrectly predicted ORFs for start codon coordinates (blue), and in the subset of false negatively predicted ORFs (red), over nine ORF length fractions (ORF length measured in nucleotides). The two subsets represent ORFs which were consistently mis-predicted by the combination of four AGEs (BASys, ISGA, RAST and xBASE) because they either had incorrectly predicted start codon coordinates, or by being a false negative predicted ORF.

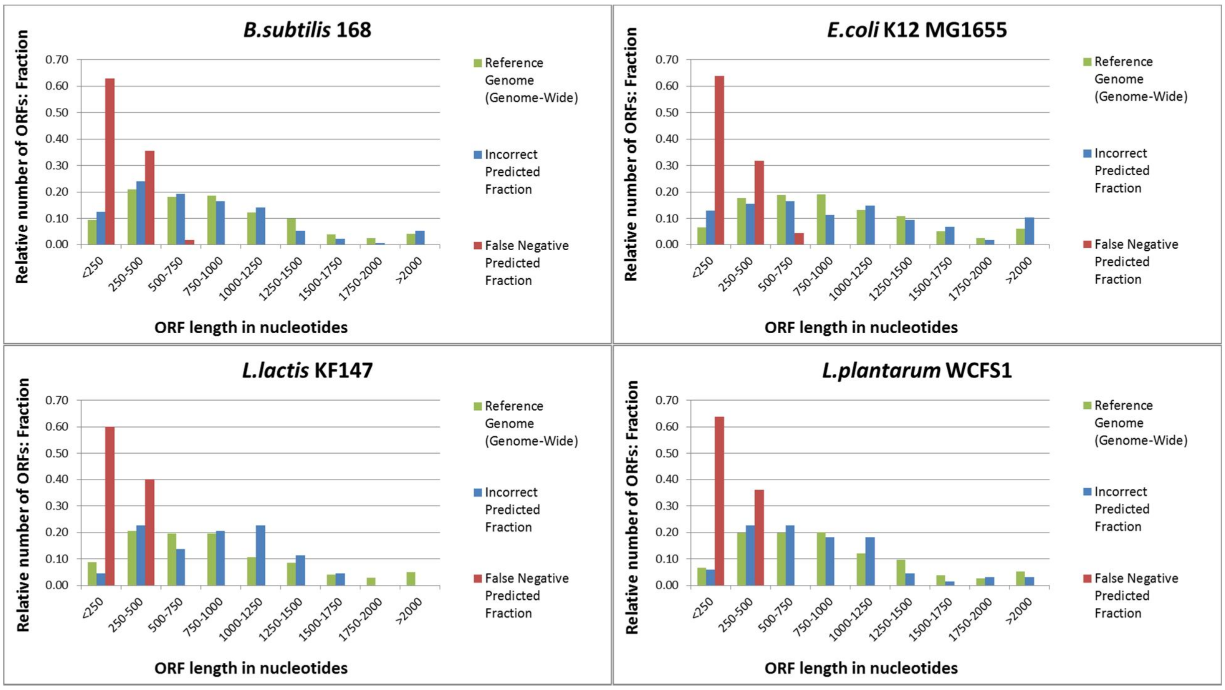

Supplement: Figure S7 — ORF length bias in false negatively predicted ORFs. (PDF) [file pone.0063523.s007.pdf]
