# Supplementary material for: Reduce Manual Curation by Combining Gene Predictions from Multiple Annotation Engines, a Case Study of Start Codon Prediction
Source: PLoS One. 2013 May 10;8(5):e63523. doi: 10.1371/journal.pone.0063523 (PMC3651085; doi:10.1371/journal.pone.0063523)
Supplement: Table S1 — Key functionalities in various AGEs. (PDF) [file pone.0063523.s008.pdf]

Table S1. Key functionalities in various AGEs.

Listed in the table below are four popular AGEs: BASys, ISGA, RAST and xBASE (listed non-alphabetically). For each engine, a selection of key features and functionalities (underlying tools) is indicated, as well as each AGE's most important underlying databases. The data was gathered from published literature and from online documentation.

Engine Name	Asgard	CodonW	CRITICA	Glimmer	Alternative ORF Predictor Available?	HMMPFAM	HOMODELLER	MAST	PredictSPTM	PROSITE	PSIPRED	RNAmmer	tRNAscan-SE	VADAR	Underlying Database ¶
**ISGA (Ergatis)**	X			X (v3)	No	X		X				X	X		RS, RT, GB, TF CO, PF, SP, CC
**RAST**				X (v3)	Yes (RAST)								X		PF, FF
**BASys**			X	X (v2.1)	No		X		X	X	X			X	RS, UP, P, KG CC, PB, PF
**xBASE (coliBASE)**		X		X (v3)	No					X		X	X		RS, UP, GB

¶ COG: CO; CyberCellDB: CC; FIGfam: FF; Genbank: GB; PDB: PB; Pfam: PF; RegTransBase: RT; SwissProt: SP; TIGRFAM: TF; UniProt: UP; UniRef: UR; RefSeq (NCBI): RS. # Asgard: metabolic pathway reconstruction; CodonW: analysis of codon and amino acid usage; CRITICA: Coding Region Identification Tool Invoking Comparative Analysis; GLIMMER: ORF prediction; HMMER: profile hidden Markov models; HMMPFAM: HMM search against the PFAM database; HOMODELLER: predicts protein 3D structures; LipoP: predicts Gram-negative lipoprotein signal peptides, other signal peptides and n-terminal membrane helices; MAST: transcription factor binding site prediction; PredictSPTM: signal peptide prediction based on HMM alignments of SignalP predicted signal peptides; PROSITE: protein motif analysis; PSIPRED: protein structure prediction server; RNAmmer: prediction of RNA-coding genes; tRNAscan-SE: tRNA prediction; VADAR: Volume Area Dihedral Angle Reporter; compilation of over 15 algorithms to assess peptide and protein structures from PDB data.
